# A Narrative Review of the Dichotomy Between the Social Views of Non-Monogamy and the Experiences of Consensual Non-Monogamous People

**DOI:** 10.1007/s10508-023-02786-1

**Published:** 2024-01-04

**Authors:** David L. Rodrigues

**Affiliations:** Iscte-Instituto Universitário de Lisboa, CIS-Iscte, Av. das Forças Armadas, 1649-026 Lisbon, Portugal

**Keywords:** Consensual non-monogamy, Mononormativity, Stigmatization, Relationship functioning, Internalized negativity

## Abstract

Monogamy is deeply rooted in most Western societies, shaping how people construe and behave in romantic relationships. These normative views facilitate the emergence of negative perceptions and evaluations when people choose not to adhere to mononormativity. Even though people in consensual non-monogamous (CNM) relationships are targets of stigmatization, research shows a dichotomy between these negative views and the relational experiences of CNM people. Indeed, people in CNM and monogamous relationships have comparable relationship functioning and quality and struggle with similar relationship problems. One of the differences is that CNM relationships afford people to explore their sexuality and fulfill their needs with multiple partners, without agreed-upon extradyadic behavior being perceived as infidelity or having deleterious consequences to relationship maintenance. These positive experiences notwithstanding, CNM people are continuously pressured by mononormativity and stigmatization, increasing the risk of internalized CNM negativity and worse personal and relational outcomes. One possible way to counteract CNM stigmatization and improve the lives of CNM people is by changing discourses surrounding non-monogamy and improving acceptance, not only in professional settings but also in the general population. Another strategy is to understand how the relationship beliefs and scripts of younger generations can help promote more inclusive and diverse societies.

## Introduction

Non-monogamy is among the most frequent sexual fantasies reported by American people (Lehmiller, 2018) and has been a popular topic of online search queries among the general public over time (Moors, 2017). Likewise, researchers across different areas have been increasingly interested in non-monogamy (Balzarini & Muise, 2020; Scoats & Campbell, 2022). Some studies suggest that about 3–7% of adults may be in a consensual non-monogamous (CNM) relationship and up to 25% may have had past experiences with consensual non-monogamy (Haupert et al., 2017a; Levine et al., 2018; Rubin et al., 2014; Séguin et al., 2017; Træen & Thuen, 2022). However, the prevalence of a monogamous norm in Western societies implies that people who depart from mononormativity are at risk of negative appraisals and struggle to accept their identity. Indeed, research has consistently shown that CNM people are socially stigmatized and perceived as having unadjusted relationships, despite ample evidence that relationship processes do not significantly differ between monogamous and CNM partners (Mogilski et al., 2023).

This narrative review offers an overview of research examining the dichotomy between the negative societal view of CNM relationships and the positive relational experiences reported by CNM people. Establishing a parallel with other sexual minorities, it also explores the personal and relational consequences of internalized negativity and offers ways to counteract CNM stigmatization. References were drawn from the consensual non-monogamies literature list, one of the education and outreach initiatives from the American Psychological Association Division 44 Committee on CNM (https://www.div44cnm.org), as well as from searches on academic databases.

## Monogamy Norms

Imagine that Jo and Sam are in a stable and committed romantic relationship. They are together for some time and then decided to have a conversation about opening their relationship to include other people. At some point, they meet Alex and decide to open their relationship. This illustrates a CNM agreement, whereby two (or more) partners agree on the possibility of having sexual encounters and/or romantic relationships with other people (e.g., open relationships, swinging, or polyamorous relationships; Conley et al., 2017; Rubin et al., 2014). This scenario, however, departs from the prototypical romantic relationship. Indeed, there is a shared belief that romantic relationships should be monogamous and partners should be sexually and emotionally exclusive to each other, conveying monogamy as better than any other type of agreement (Conley et al., 2012b). These beliefs are imposed through socialization (Conley et al., 2013; Henrich et al., 2012; Ryan & Jetha, 2012), shared in political, public, and religious discourses (Cardoso et al., 2021), and even shared by some experts and professionals (Grunt-Mejer & Chańska, 2020; Herbitter et al., 2021). This belief is so pervasive that partners often assume monogamy (Muise et al., 2021) and rely on their partner’s exclusivity (Conley et al., 2017; Ziegler et al., 2015). And yet, most partners fail to address the topic of monogamy in their relationships. For example, Badcock et al. (2014) found that more than 96% of participants expected their partner and themselves not to have sex with other people, but only about 30% explicitly addressed these expectations with their partner.

This lack of clear communication about monogamy expectations opens the possibility that partners have different conceptions about extradyadic behaviors and infidelity. For example, people can engage in behaviors perceived as infidelity by their partners (e.g., watching pornography alone), even though they consider otherwise and believe their relationship to be monogamous (Liu & Zheng, 2019). In their study with users from a dating website, Rodrigues et al. (2017a) found that even though users reported being monogamous, more than 88% registered alone on the website and more than 66% had sex with another user. Aligned with the argument that monogamy expectations shape infidelity beliefs, the authors also found that users who have enacted extradyadic sex believed that ambiguous (e.g., talking with another person in secret) and explicit behaviors (e.g., sexual intercourse) were less indicative of infidelity when compared to users who did not enact extradyadic sex. No difference between groups was observed in the perception of deceptive behaviors (e.g., lying to the partner). These different conceptions and behaviors are likely to result in relational conflicts and problems.

Regardless of what monogamy expectations imply for behaviors and relationship quality, the negative perceptions and reactions that monogamous people have when they are faced with infidelity (de Visser et al., 2020; Kruger et al., 2015; Previti & Amato, 2004) parallel those shared by most Western societies. Indeed, extradyadic behaviors (particularly those involving sexual activity) and people who enact these behaviors tend to be socially condemned, regardless of whether or not romantic partners consensually agree upon those behaviors.

## Consensual Non-Monogamy Stigmatization

Research has consistently shown a negative appraisal of non-monogamy. In a series of studies, Conley et al. (2013) asked about the benefits of having a monogamous relationship. Participants indicated relational benefits (e.g., committed relationships, trusting another person, having a meaningful connection with another person, or having a family) but also health and moral benefits. When asked to make judgments about monogamous and CNM relationships, participants reported more negative perceptions about the relational dynamics of CNM relationships (e.g., less comforting, trusting, and intimate relationships; partners have sex with each other less frequently), and at the same time perceived CNM partners more negatively in arbitrary traits (e.g., less likely to floss daily; less invested in taking care of others; less caring, reasonable, and satisfied with life). In another study, Grunt-Mejer and Campbell (2016) compared different relationship structures and found that monogamous partners were perceived as the most satisfied with their relationship, the most moral, and with the highest cognitive abilities (e.g., more intelligent). In contrast, monogamous partners who wanted to maintain their relationships but enacted extradyadic behaviors (named “cheating” by the authors), received the most negative appraisals. The negative halo surrounding non-monogamy has been replicated in other studies. For example, Balzarini et al. (2018) found that monogamous participants reported wanting more social distance from partners who were in open, swinging, or polyamorous relationships. In another study, Rodrigues et al. (2022) found that monogamous partners were perceived as the most trustworthy, moral, committed, and sexually satisfied, whereas CNM partners were perceived as the most promiscuous and likely to have sexually transmitted infections.

The stigmatization of non-monogamy has also been extended to other phenomena with potentially more severe consequences. For example, there is evidence that CNM people are dehumanized (Rodrigues et al., 2018, 2021b). Broadly, dehumanization occurs when people are deprived of certain attributes that are uniquely human and not shared with objects or animals (Haslam, 2006). This phenomenon is observed among people from different countries, different ethnic groups, or different social groups (Haslam & Loughnan, 2014). People dehumanize others by perceiving them to lack secondary emotions that are uniquely human and require a higher level of processing (e.g., love, embarrassment), and instead experience mostly primary emotions that are shared with other animals and require a lower level of processing (e.g., anger, happiness; Demoulin et al., 2004; Leyens et al., 2000, 2001; Vaes et al., 2012). Dehumanization has been associated with negative psychological and physical consequences, such that dehumanized people tend to be victims of verbal and physical abuse in different contexts (e.g., Rai et al., 2017), including in romantic relationships (Pizzirani & Karantzas, 2019; Pizzirani et al., 2019). In a study that included samples from Portugal, Italy, and Croatia, Rodrigues et al. (2018) found that participants attributed more secondary (vs. primary) emotions to monogamous partners, and more primary (vs. secondary) emotions to CNM partners. Interestingly, these findings were independent of whether partners were described as heterosexual or gay (see also Moors et al., 2013; Rodrigues et al., 2022), suggesting that departures from mononormativity were more salient in determining stigmatization, so long partners were committed to a monogamous relationship. In a follow-up study, Rodrigues et al. (2021b) extended these findings by showing that dehumanization occurred because participants perceived CNM (vs. monogamous) partners as more immoral and less committed to their relationship. Taken together, these findings indicate that the stigmatization of CNM people (and their relationships) is mostly anchored on departures from socially conveyed norms of emotional exclusivity and morality. The stigma experienced by CNM people sharply contrasts with their relational experiences.

## Personal Experiences with Non-Monogamy

Research has highlighted some a priori demographic differences between monogamous and CNM people (e.g., gender, sexual orientation; Balzarini et al., 2019b; Haupert et al., 2017b; Moors et al., 2021a; Stults, 2019). Regardless, other individual variables have been shown to shape how people pursue their affective and sexual needs. For example, people with a more unrestricted sociosexuality (i.e., predisposed to have multiple sex partners) in a monogamous relationship are more likely to experience relationship distress (e.g., Webster et al., 2015), enact extradyadic behaviors (e.g., Barta & Kiene, 2005; Rodrigues & Lopes, 2017; Rodrigues et al., 2017b) and have their relationship end (e.g., French et al., 2019). Although there is some degree of assortative mating in sociosexuality (Manning, 2006), this does not mean that romantic partners with unrestricted sociosexuality are at odds with relationship failure. For example, Rodrigues and Lopes (2017) found that partners with a more unrestricted sociosexuality were more likely to have enacted extradyadic sex in their current relationship if they reported lower (but not higher) relationship commitment. Using a dyadic approach, Markey and Markey (2013) found that partners with a more restricted sociosexuality were the most committed, followed by partners with a more unrestricted sociosexuality. In contrast, the lowest relationship commitment was reported by partners with unmatched sociosexuality. Hence, having a partner with similar predispositions in sexual behavior and desires might help to discuss alternative ways to accommodate affective and sexual needs. Unsurprisingly, then, CNM (vs. monogamous) partners tend to have a more unrestricted sociosexuality (Mogilski et al., 2020), and those who act upon their unrestricted sociosexuality tend to be more satisfied and committed in their relationship, and report better quality of life (Rodrigues et al. 2016, 2017b, 2019b).

Research has also suggested that CNM people may be better equipped to express intimacy and feel closer to their partners. For example, Cohen (2016) and Wood et al. (2021) found that CNM people highlight the ability to experience new things, the freedom to explore sexuality and sexual satisfaction, how close they feel to their partner, and need fulfillment as some of the positive experiences and motives for engaging in CNM relationships. In contrast, having to deal with social stigma, jealousy, and trust issues were among the negative aspects related to a CNM relationship. Some of these benefits are unique to non-monogamy (e.g., personal growth, need fulfillment), whereas others are shared with monogamy (e.g., stable relationship, love; Moors et al., 2017). Aligned with this, Murphy et al. (2021) found that partners who decided to engage in non-monogamy experienced significant increases in sexual satisfaction later on, despite not reporting changes in relationship quality. In other words, CNM partners likely fulfilled specific needs with other partners that were being unmet in their current relationship.

When negotiating non-monogamy, CNM partners also rely on open communication to mutually establish and clarify the boundaries of their agreement (Andersson, 2022; Cohen, 2016; Wood et al., 2021). Open communication promotes perceptions of equity in the relationship, trust in one another, and commitment (Hangen et al., 2020; McLean, 2004; Moors et al., 2015, 2017), helps to work around jealousy issues (de Visser & McDonald, 2007), and may help CNM partners who are not comfortable to take a step back and discuss the terms of the agreement (e.g., Philpot et al., 2018). When comfortable with their agreement, CNM partners report levels of affection, eroticism, and relationship functioning (e.g., satisfaction, commitment, trust, intimacy) that are comparable (if not higher) than those reported by monogamous partners (Balzarini et al., 2019c; Lecuona et al., 2021; Mogilski et al., 2017; Rodrigues et al., 2017b; Rubel & Bogaert, 2015). CNM (vs. monogamous) partners are also likely to use more positive strategies to solve relational issues (e.g., more problem solving; less withdrawal in conflicts) and report more well-being (Brooks et al., 2022). Other studies have shown that CNM people are equally or more sexually satisfied than monogamous partners (Conley et al., 2018; Parsons et al., 2012; Rodrigues et al., 2021a), particularly when both CNM partners fulfill and are responsive to each other’s needs (Muise et al., 2019). They also tend to experience relatively low levels of jealousy and can even feel good (i.e., compersion) when their partner has extradyadic relationships (Balzarini et al., 2021; Barker, 2005; Ritchie & Barker, 2006). And even though CNM people tend to be more focused on their sexual novelty, pleasure, and sexual satisfaction when compared to monogamous people, emotional motives are similar in both relationships (Mitchell et al., 2020; Wood et al., 2018). Still, CNM people tend to be particularly careful with their own and their partners’ sexual health, especially when compared to monogamous people engaged in infidelity (Conley et al., 2012a; Lehmiller, 2015), arguably because CNM people perceive to have more self-control in sex (Rodrigues et al., 2019a, 2019c).

Consensual non-monogamous people do not necessarily perceive extradyadic behaviors as infidelity insofar as both partners stick to their agreement, unlike monogamous people. For example, Cohen (2016) found that CNM people consider behaviors such as lying or withholding information from the partner as more indicative of infidelity and tend to be more lenient about explicit behaviors (e.g., extradyadic sex), which are typically conceived as infidelity by monogamous people. Like monogamous people, however, CNM people experience negative emotions if their agreement is crossed. For example, Mogilski et al. (2019) found that CNM participants were more distressed and experienced greater jealousy after imagining their partner establishing an emotional bond with a person outside of the boundaries mutually agreed upon. However, these similarities may be particularly true when CNM people are considering their primary partners. Indeed, the authors found that CNM people were more confident that their primary (vs. secondary) partner would not engage in infidelity, were more distressed when thinking about that possibility, and were more protective of their primary relationship. In another study, Mogilski et al. (2017) found no differences between CNM and monogamous people in relationship satisfaction, but only if CNM people were considering their primary partner. CNM people had also been in a relationship with their primary (vs. secondary) partner for a longer period, viewed this partner as a more desirable long-term mate, and were more likely to discuss and downplay extradyadic sexual experiences with them. Extending these findings to include social perceptions, Balzarini et al. (2017, 2019a, 2019c) found that polyamorous partners with a primary-secondary relationship structure reported greater more relationship quality toward primary partners (e.g., more commitment, satisfaction, love, and attraction; better communication) and perceived their primary relationships to be more socially accepted (e.g., by friends and family). In contrast, polyamorous partners spent more time on sexual activity with secondary partners, but they were also more secretive about these secondary relationships. Despite being smaller in magnitude, these differences also emerged among polyamorous partners who rejected hierarchical labels in their relationship. In some ways, then, it seems that societal views may restrict and determine how CNM people perceive and behave in their relationships.

## Challenges for Consensually Non-Monogamous People

Even though CNM people have adjusted and functioning relationships, they still must cope with the constant exposure to monogamous values and expectations. Exposure to normative views can lead to the internalization of such norms, create internal conflicts, and have consequences for relationships, health, and well-being. This reasoning follows research framed by the minority stress model (Meyer, 1995), which has shown that people from sexual minorities (e.g., LGBTQIA + people) face unique stressors in response to context cues (e.g., normative pressure to conform), which can result in internalized negativity (Rostosky & Riggle, 2017) and poorer health (Dürrbaum & Sattler, 2020). For example, Torres and Rodrigues (2022) found that Portuguese and Turkish gay men who endorsed more heteronormativity beliefs also reported more internalized homonegativity. Such negative experience has been associated with a negative self-identity (Riggle et al., 2014), discomfort with one’s sexual orientation and fear of coming out (e.g., identity concealment; Dyar et al., 2018), worse relationship functioning (Doyle & Molix, 2021), and riskier behaviors in sex (Burton et al., 2020).

Much like other sexual minorities, CNM people are met with stigmatization daily, either by being continuously exposed to monogamy expectations, exposed to the stigmatization of other CNM people, or directly confronted for their non-adherence to monogamy after assuming their relationship configuration. Being confronted with stigmatization leads CNM people to question whether or not to disclose their CNM identity and relationship configuration to others (Valadez et al., 2020), trust the healthcare system to address their specific health needs (Vaughan et al., 2019), or maintain their therapeutic relationship after seeking for psychological help (Schechinger et al., 2018). This stigmatization can also result in internalized negativity and worse outcomes (e.g., psychological distress; Mahar et al., 2022). Aligned with this reasoning, Moors et al. (2021a) found that CNM people who were uncomfortable with non-monogamy (i.e., endorsed more internalized CNM negativity) were less satisfied with their current relationship agreement, and reported being less satisfied and committed to their primary partner. Extending these findings, Rodrigues et al. (2023) found that CNM people who endorsed more mononormative beliefs reported more internalized CNM negativity, had a more negative view about themselves (i.e., reported experiencing negative emotions more often and positive emotions less often), and perceived their partners as more immature, unrefined, exploitable, and emotionless (i.e., were more likely to dehumanize their partner).

There is still a restricted understanding of the consequences of internalized CNM negativity on health and well-being, particularly its pervasiveness in the CNM community and the extensiveness of its effects over time. Still, researchers can consider variables that may help counter stigmatization and improve the experiences of CNM people.

## A Brighter Future

Different strategies can help improve intergroup relations and decrease intergroup bias (Boin et al., 2021; Eisenberg et al., 2010; Gonzalez et al., 2015; Visintin et al., 2020). One possibility is to improve knowledge among professionals and the general public. On the one hand, therapists with more informed, affirming, and inclusive approaches to non-monogamy are better equipped to work with, and to be perceived as helpful by their CNM patients (Schechinger et al., 2018). On the other hand, people with more positive CNM attitudes are less likely to stigmatize CNM partners (Rodrigues et al., 2021b). For example, Rodrigues et al. (2022) showed that having more positive CNM attitudes was associated with less stigmatization, because participants perceived CNM (vs. monogamous) partners to be more open to change. In contrast, having more negative CNM attitudes was associated with more stigmatization because participants perceived CNM (vs. monogamous) partners to be less conservative and more open to change. In other words, favorable CNM views can improve acceptance by attributing strength to mononormativity departures, whereas unfavorable CNM views can foster negative appraisals through the lens of traditionalism and mononormative disruption.

Perceptions of CNM relationships may also benefit from changes in the way multi-partner relationships are perceived and enacted by younger adults (Hamilton & Winward, 2022), particularly among those who consider this to be a period of exploration and trying new things (Olmstead & Anders, 2022). For example, Sizemore and Olmstead (2018) found that one in four young adults was willing or open-minded about the possibility of having a CNM experience, and Stephens and Emmers-Sommer (2020) found that 48% of younger people were in a CNM relationship (i.e., monogamist, negotiable, open relationship, swinging, polyamorous, or other). Possibly, experimenting with alternative relationship structures and openly talking about their experiences with close others (e.g., friends, prospective partners) may be increasingly recurring among younger generations. Aligned with this reasoning, younger adults are more open to experimenting with sexuality and sexual relationships, and more predisposed to have multiple casual sexual relationships with different partners (Alvarez et al., 2023; Claxton & van Dulmen, 2013; James-Kangal et al., 2018; McMahan & Olmstead, 2021; Sizemore & Olmstead, 2018). Similar to the perceived benefits and behaviors reported by CNM partners, younger people engage in different casual sexual relationships (e.g., friends with benefits, fuck buddies, one-night stands) because these relationships afford them the freedom to explore sexuality, excitement, and novelty, provide sexual and/or affective intimacy, and help them fulfill multiple needs with distinct partners, without discarding the importance of sexual health and protection (Alvarez et al., 2021; Luz et al., 2022). If younger generations are becoming less attached to the mononormative views of romantic relationships, then they should be more open to defying mononormativity as the prevalent social norm and be more accepting of alternative relationship configurations.

## Conclusion and Implications

This narrative review highlighted some of the ways through which mononormativity fosters CNM stigmatization while contributing to a more comprehensive understanding of relationship science. From theoretical and methodological standpoints, the evidence herein reviewed can have implications for the way researchers think about relationship processes (Hammack et al., 2019), particularly because monogamy tends to be assumed by several theoretical perspectives and the assessment of relationship configuration is often overlooked in studies. For instance, both the interdependence theory (Arriaga, 2013) and the investment model (Rusbult et al., 2012) assume monogamy when discussing the implications of perceived comparison levels and the perceived quality of potential alternative partners for relational outcomes. Self-expansion theory (Aron et al., 2022) assumes monogamy when discussing the implications of developing a common self with the partner for relationship stability and quality. Likewise, the sexual communal strength framework (Muise & Impett, 2016) assumes monogamy when examining the implications of being responsive to the partner’s sexual needs. By contrasting social views and personal experiences, this review can potentially contribute to revising and extending established theoretical frameworks to include CNM relationships (Impett et al., 2020; Lee & O’Sullivan, 2019; MacDonald et al., 2021; Wood et al., 2021) or inform the extension of theoretical perspectives already acknowledging sexual and gender diversity in partnered sexuality (Abed et al., 2019).

Moving forward, researchers should strive to acknowledge *how*, *why*, and *under which conditions* relationship agreements and configurations are negatively perceived and shape the way people navigate their lives and relationships. These efforts can potentially contribute to open discussions and changing discourses toward the acceptance, affirmation, and celebration of relationship diversity.

## Data Availability

Not applicable.
